# Antimicrobial Activity of Novel Ni(II) and Zn(II) Complexes with (E)-2-((5-Bromothiazol-2-yl)imino)methyl)phenol Ligand: Synthesis, Characterization and Molecular Docking Studies

**DOI:** 10.3390/antibiotics12111634

**Published:** 2023-11-17

**Authors:** Inas Al-Qadsy, Waseem Sharaf Saeed, Ahmad Abdulaziz Al-Owais, Abdelhabib Semlali, Ali Alrabie, Lena Ahmed Saleh Al-Faqeeh, Mohammed ALSaeedy, Arwa Al-Adhreai, Abdel-Basit Al-Odayni, Mazahar Farooqui

**Affiliations:** 1 Chemistry Department, Maulana Azad College of Arts, Science and Commerce, Aurangabad 431001, India; 2 Department of Restorative Dental Sciences, College of Dentistry, King Saud University, P.O. Box 60169, Riyadh 11545, Saudi Arabia; wsaeed@ksu.edu.sa (W.S.S.);; 3 Chemistry Department, College of Science, King Saud University, P.O. Box 2455, Riyadh 11451, Saudi Arabia; 4 Groupe de Recherche en Écologie Buccale, Faculté de Médecin Dentaire, Université Laval, Quebec, QC G1V 0A6, Canada; 5 Microbiology Department, Dr. Babasaheb Ambedkar Marathwada University, Aurangabad 431004, India; lenaalfaqeeh8@gmail.com

**Keywords:** Schiff base, (E)-2-((5-bromothiazol-2-yl)imino) methyl) phenol, microwave-assisted synthesis, nickel complex, antibacterial activity, antibiotic Streptomycin, *E. coli* NAD synthetase, docking study

## Abstract

In order to address the challenges associated with antibiotic resistance by bacteria, two new complexes, Ni(II) and Zn(II), have been synthesized using the conventional method based on Schiff base ligand (E)-2-((5-bromothiazol-2-yl) imino) methyl) phenol. The Schiff base ligand (HL) was synthesized using salicylaldehyde and 5-(4-bromophenyl)thiazol-2-amine in both traditional and efficient, ecologically friendly, microwave-assisted procedures. The ligand and its complexes were evaluated by elemental analyses, FTIR spectroscopy, UV-Vis spectroscopy, nuclear magnetic resonance (NMR), thermogravimetric analysis (TGA) and magnetic susceptibility. The ligand and its complexes were tested for antibacterial activity against three Gram-positive bacteria (*Staphylococcus aureus* ATCC 25923, *Methicillin-resistant Staphylococcus aureus* ATCC 43300 and *Enterococcus faecalis* ATCC 29212) and three Gram-negative bacteria (*Pseudomonas aeruginosa* ATCC 27853, *Escherichia coli* ATCC 25922 and *Klebsiella pneumoniae* ATCC 700603). The findings demonstrate the potent activity of the ligand and its complexes against selective bacteria but the Ni(II) complex with MIC values ranging from 1.95 to 7.81 µg/mL outperformed all other compounds, including the widely used antibiotic Streptomycin. Furthermore, the docking study provided evidence supporting the validity of the antimicrobial results, since the Ni complex showed superior binding affinity against to *E. coli* NAD synthetase, which had a docking score (−7.61 kcal/mol).

## 1. Introduction

One of the most serious problems facing different parts of the world is increased antibiotic-resistant bacteria and microbial infections, which have contributed to increased diseases and fatalities in recent years. Antibiotics are chemical substances that either kill or prevent the growth of microorganisms. They also find extensive application in the treatment of bacterial infections in both humans and animals. Additionally, they are utilized in nonmedical contexts, such as motivating livestock growth. However, bacteria have become more resistant to various antibiotics, so scientists are working to develop new, more powerful antibiotics to control this problem [1,2,3]. Heterocyclic compounds, including thiazoles and their derivatives, are highly regarded for their extensive range of uses. Consequently, scientists are devoting increased attention to exploring the potential of thiazole molecules [4,5]. A wide variety of medications contain the thiazole nucleus, including those that are antimicrobial and antifungal [6,7,8,9], anti-inflammatory [10], anticancer [11], antihypertensive [12], anti-HIV [13], antidiabetic [14], a scaffold hopping [15] scaffold-based ratiometric fluorescent probe [16], and those that treat *Helicobacter pylori* and *Mycobacterium tuberculosis* [17].

A series of α-thiazolyl aminomethylene bisphosphonates exhibited promising antibacterial activity against gram-positive (*Bacillus subtilis*) and gram-negative (*Escherichia coli*, *Pseudomonas aeruginosa*) bacteria as compared to the standard drug streptomycin [18]. Several studies have shown that thiazole Schiff bases are very effective at killing bacteria, including both pathogenic gram-positive and gram-negative bacteria. This activity is attributed to the presence of functional groups in the thiazole Schiff base molecule that interact with bacterial cell walls or disrupt bacterial metabolic processes [19]. Moreover, the simplicity and ease of synthesis of thiazole Schiff bases and the possibility of modifying their chemical structure provide opportunities for the development of new compounds with enhanced antibacterial properties. Therefore, many researchers have converted aminothiazole to Schiff bases as a single framework to increase activity, such as 4-(6-Fluoro-1,3-benzothiazol-2-yl)amino-2-(2-chlorophenyl-methylidene)amino-1,3-thiazole, (4-(6-Fluoro-1,3-benzothiazol-2-yl)amino-2-(2-nitrophenyl-methylidene)amino-1,3-thiazole, 4-(6-Fluoro-1,3-benzothiazol-2-yl)amino-2-(3,4-dimethoxyphenyl-methylidene)-amino-1,3-thiazole and 4-(6-Ethoxy-1,3-benzothiazol-2-yl)amino-2-(2-chloro-phenyl-methylidene)amino-1,3-thiazole. All of these Schiff bases have antifungal, antibacterial and anthelmintic actions [20]. When benzothiazole is linked to thiophene-2-carbonyl chloride through an amide bond, it can be used as a model for antimicrobial activity, such as N-(6-chloro-benzothiazol-2-yl) thiophene-2-carboxamide [21]. The Schiff base, prepared from 4-(4-(4-Bromophenyl) thiazol-2-ylimino)methyl)-2-methoxyphenol, works well against *S. aureus* and *E. coli*, but N-(3,4,5-Trimethoxybenzylidene)-4-(4-bromophenyl)thiazol-2-amine was cytotoxic against *B. subtilis* [22]. Some thiazole–pyrazoline Schiff base hybrid derivatives display significant antibacterial activity, as shown by the finding that 2-(1-((E)-(2-thiazol-4-yl)phenol)methyl)-4,5-dihydro-3-(4-nitrophenyl)-1H-pyrazol-5-yl)phenol exhibited higher activity than the standard drug amoxicillin against *P. aeruginosa* and *E. coli*, while the other derivatives had a modest to negligible antibacterial effect [23].

In addition to the Schiff bases that were synthesized from thiazole, their complexes are also more biologically effective than their parental free ligands. A Zn(II) complex was derived from two bioactive Schiff bases that were synthesized from 5-nitro-2-aminobenzothiazole or 5-nitro-2-aminothiazole with 5-chloroisatin, since the Zn(II) complex exhibited more effective antibacterial, antioxidant and cytotoxic activities than free ligands [24]. A novel Schiff base that was synthesized from 2-amino-4-phenyl-5-methyl thiazole and 4-(diethylamino)salicyaldehyde was used to create new Co (II), Ni (II), Cu (II) and Zn (II) complexes. An anticancer study found that the Zn (II) complex significantly inhibited the growth of HepG2, MCF7, A549 and HCT116 cell lines [25]. Diorganotin (IV) complexes of Schiff bases synthesized from 4-(4-bromophenyl)thiazol-2-amine and salicylaldehyde derivatives have shown promising antibacterial activity against two gram-positive bacteria (*Bacillus cereus* and *Bacillus subtilis*) and two gram-negative bacteria (*Escherichia coli* and *Pseudomonas aeruginosa*). This activity is attributed to their ability to interact with microbial cell membranes and disrupt their integrity [19]. Based on salicylidene-4-chlorophenyl-2-aminothiazole, Ni (II), Cu (II) and Cd (II) complexes were synthesized. The antibacterial tests indicate that the cadmium complex is more active than the nickel (II) and copper (II) complexes [26]. Ni(II) and Zn(II) complexes were synthesized using the Schiff base 2-(4-(diethylamino)-2-hydroxybenzylidene)-N-(4-phenylthiazol-2-yl)hydrazinecarboxamide; when assessed for their activity against bacteria (*S. aureus* and *E. coli*) and fungi (*A. flavus* and *A. niger*), the result showed exceptional effects [27].

Given how important thiazole is, in the current work, the thiazole ligand (E)-2-((5-bromothiazol-2-yl)imino)methyl)phenol (HL) was synthesized by a conventional method and a microwave technique from 5-(4-bromophenyl)thiazol-2-amine with salicylaldehyde. However, a microwave will improve yield, reduce processing times, use a safe solvent (water and PEG) and leave no trace of pollution. Moreover, new Ni(II) and Zn(II) metal complexes were effectively synthesized from (E)-2-(((5-bromothiazol-2-yl)imino)methyl)phenol and metal salt ZnCl_2_ or Ni(NO_3_)_2_.6H_2_O. The HL ligand and their complexes are characterized using a variety of analytical techniques, including thermal analysis, magnetic moment and spectroscopy methods such as NMR, FTIR and UV-visible. Additionally, it would be intriguing to look into the biological effects that this new ligand and its complexes have on some gram-positive (*Staphylococcus aureus* ATCC 25923, *Methicillin-resistant Staphylococcus aureus* ATCC 43300 and *Enterococcus faecalis* ATCC 29212) and gram-negative bacteria (*Pseudomonas aeruginosa* ATCC 27853, *Escherichia coli* ATCC 25922 and *Klebsiella pneumoniae* ATCC 700603). Moreover, molecular docking studies are applied to the thiazole ligand and its complexes to support the in vitro result.

## 2. Results

### 2.1. Synthesis of Schiff Base Ligand

**Scheme 1:**
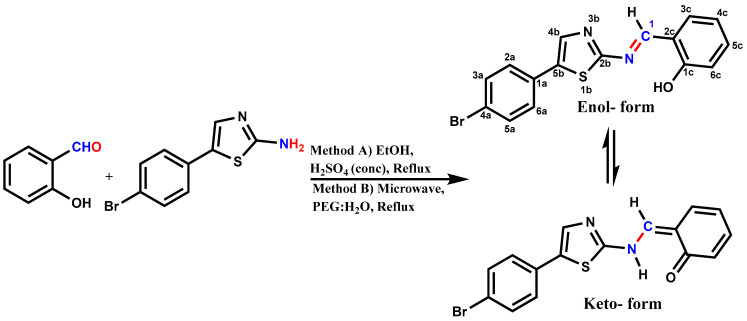
Schematic presentation of (E)-2-(((5-bromothiazol-2-yl)imino)methyl)phenol ligand.

During the successful synthesis of the Schiff base ligand (E)-2-((5-bromothiazol-2-yl)imino)methyl)phenol (HL) from 5-(4-bromophenyl)thiazol-2-amine and salicylaldehyde, as illustrated in Scheme 1, the Schiff base was synthesized using the conventional method by following the steps of [28] with some modifications. In addition, the Schiff base was prepared utilizing microwave-assisted techniques with some alterations to the [29] protocol, as described in Scheme 1. Elemental analysis, FTIR, ^1^H NMR, ^13^C NMR, UV-Vis and thermal analysis were used to confirm the structure of thiazole Schiff bases.

The microwave method A significantly reduced the reaction time compared to conventional method B (which used ethanol as a solvent and had a reaction time of 6 h), whereas the microwave technique needs a three-minute reaction time and was carried out in a 1:1 combination of water with PEG, which is unquestionably an environmentally friendly solvent. Furthermore, the yield from the microwave-assisted strategy was 96% was greater than the method A (89%), suggesting that the novel method is considered an efficient method and may be utilized for thiazole Schiff base synthesis. The color of the ligand is greenish yellow, while the strong H-bonding between the hydroxyl group and imin group may be the cause of the melting point being quite high at 196 °C. 

**Figure 1:**
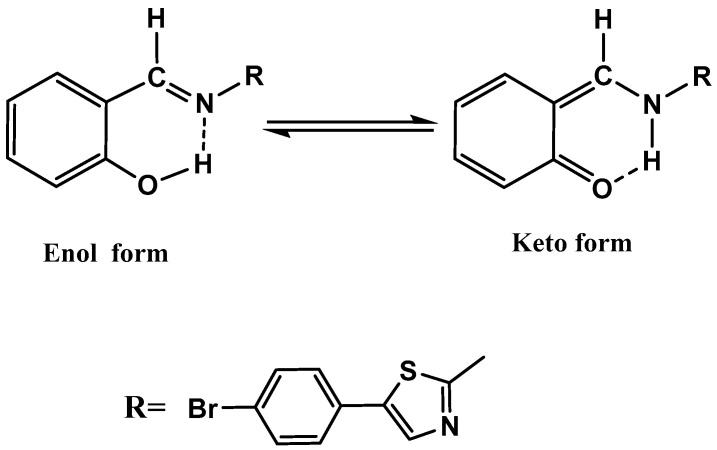
Intramolecular hydrogen bonding in thiazole ligand.

The thiazole ligand’s structure might show the formation of the keto amine tautomer. Scheme 1 illustrates how stabilization happens when a proton of hydroxyl is transferred to the amine group’s N atom, where the enol form stabilizes in solution and the keto form stabilizes in solid state [30]. As can be seen in Figure 1, Schiff base compounds generated from aromatic aldehydes contain a hydroxyl group in position 2 of the aldehyde group; intramolecular H-bonding is frequent [31].

### 2.2. Synthesis of Metal Complexes

**Scheme 2:**
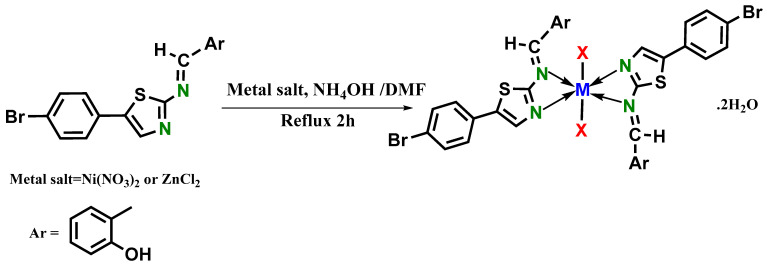
Synthesized reaction of Ni(II) and Zn(II) of (E)-2-(((5-bromothiazol-2-yl)imino)methyl) phenol.

The thiazole ligand was used to synthesize novel metal complexes of Ni(II) and Zn(II) using DMF as a solvent and ammonia solution as a basic medium, as indicated by Scheme 2. The resulting colored solution was poured onto ice, filtered precipitate and dried at room temperature, then in a vacuum oven. Interpretation CHN analysis, electronic spectra, FTIR spectra, magnetic moment and TGA thermal analysis were used to suggest the chemical structure of Ni(II) and Zn(II) complexes that have octahedral geometry.

### 2.3. Elemental Analysis

The CHN elements of the thiazole ligand and their complexes were measured using the CHNSO analyzer, and the findings are shown in Table 1. The measured and calculated percentages of C, H, N, and S atoms are extremely close, indicating a good ligand and complex synthesis.

### 2.4. ^1^H NMR Analysis

Figure S1 displays the integrated peaks for all protons in the thiazole ligand from ^1^H NMR in dimethyl sulfoxide (DMSO-d_6_) as the solvent and tetramethylsilane (TMS) as the reference, and these peaks are described below.

A singlet peak belonging to the hydrogen of the hydroxyl group was observed at δ 10.72 ppm, indicating a stable enolic form in the solution, while another singlet peak corresponding to the hydrogen of the new imine group of Schiff base was detected at δ 9.70 ppm. The hydrogen of the thiazole ring emerges as a singlet peak at δ 7.22 ppm but the protons of the aromatic rings appeared as multiple peaks in the range δ (6.80–7.55) [19,32].

### 2.5. ^13^C NMR Analysis

The ^13^C NMR spectrum of the thiazole ligand in Figure S2 shows the chemical shift in all-equivalent and non-equivalent carbon atoms, which corresponds to the structure. Since carbon 2b in the thiazole ring is attached to two nitrogen atoms and one sulfur atom, which has a chemical shift of 166.4 ppm, it is more deshielded than the two carbons 4b and 1, attributed to the thiazole ring and the imine Schiff base. Both carbons show a single peak at chemical shifts of 154.3 and 158.8 ppm, respectively [33,34].

Furthermore, the carbons linked to hydroxyl groups and the sulfur atoms 1c and 5b are less shielded, resulting in single peaks at δ 160.8 ppm and 141.9 ppm, respectively. However, the peaks in the chemical shift range (115.7–132.7) ppm are ascribed to shielded carbon atoms [19,22,35].

### 2.6. FTIR Analysis

**Figure 2:**
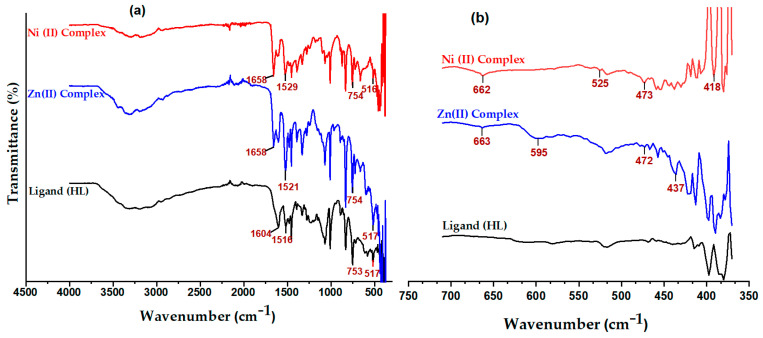
FTIR spectra (**a**,**b**) of (E)-2-(((5-bromothiazol-2-yl)imino)methyl)phenol ligand and its Ni(II), Zn(II) complexes.

The FTIR spectra of the thiazole ligand (HL) and its complexes are given in Figure 2a. The appearance of a new band at 1604 cm^−1^ reflects a newly formed azomethine group *v*(HC=N); this observation is further supported by the disappearance of both the primary amine group and the aldehyde group. Due to the coordination of the imine group with the metal ion, its absorption band shifts to a higher frequency.

The band at 1516 cm^−1^ was assigned for *v*(C=N) of the thiazole ring in the HL ligand, which shifted to a higher frequency for Zn(II) and Ni(II) complexes and was seen at 1521 cm^−1^ and 1529 cm^−1^, respectively. These shifts suggest that the thiazole nitrogen atom is involved in coordination with metal ions. Schiff-base compounds that are derived from aromatic aldehydes and have a hydroxyl group in the second position from the aldehyde group often have a tendency to form hydrogen bonds. As a result, the free HL ligand displays broadband within the 3442–3062 cm^−1^ range, which is indicative of intramolecular hydrogen bond interactions. This is illustrated in Figure 1 [35,36,37]. A broad band was also seen in their complexes due to the formation of a hydrogen bond between the OH group with water include in the complexes, and the fact that the stretching vibration of C-O did not change more between the complexes and the free ligand showed that the OH group is not involved in the coordination with metal ions. In the free ligand, the *v*(C-S-C) band assigned at 753 cm^−1^ had very little effect on complexes, revealing that the sulfur atom does not participate in the coordination. In the complexes’ spectra, new bands were observed at the lowest frequency in the range 663–418 cm^−1^, corresponding to *v*(M-N), as shown in Figure 2b [35,36].

### 2.7. Electronic Spectra and Magnetic Moment

**Figure 3:**
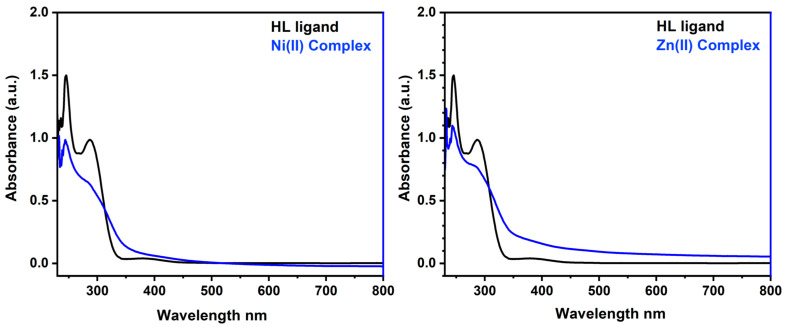
Electronic spectra of (E)-2-(((5-bromothiazol-2-yl)imino)methyl)phenol ligand, Ni(II) complex and Zn(II) complex.

The electronic spectra of the synthesized thiazole ligand (HL) and its complexes were taken in DMSO: H_2_O solution (2:8) by volume in the wavelength range 200–800 nm at room temperature, as represented in Figure 3. The electronic spectra of the HL ligand contain peaks at (267–286) nm in the ultraviolet area that are attributed to π–π* of phenyl and the thiazole ring. The band almost stays in position after complexation, confirming that the π electron did not take part in the complexation reaction. In contrast, weak peaks associated with n–π* transitions were seen in the visible region at wavelengths of 379 nm, 541 nm, and 543 nm of (>C=N) azomethine and thiazole groups that changed during complexation, which is evidence that the coordination involved a nitrogen atom [19,38].

While, at 706 nm (*v*_1_), the nickel complex shows only one weak d-d transition band, which is ascribed to ^3^A_2g_(F)→^3^T_2g_(F), other weak bands could be seen at (310–364) nm for n–π [39]. The measured magnetic moment value for the nickel (II) complex is 3.2 BM, which falls within the predicted range of 2.8–3.5 BM, revealing that it contains an octahedral environment [35,36].

The electronic spectra of the zinc complex include weak bands for n–π* and MLCT at (340–387) nm and the magnetic moment of the Zn(II) complex is diamagnetic [40,41].

### 2.8. Thermal Analysis

The thermogravimetric technique was utilized to evaluate the new metal complexes’ thermal stability and the number of water molecules present outside the inner coordination sphere (crystalline water).

**Figure 4:**
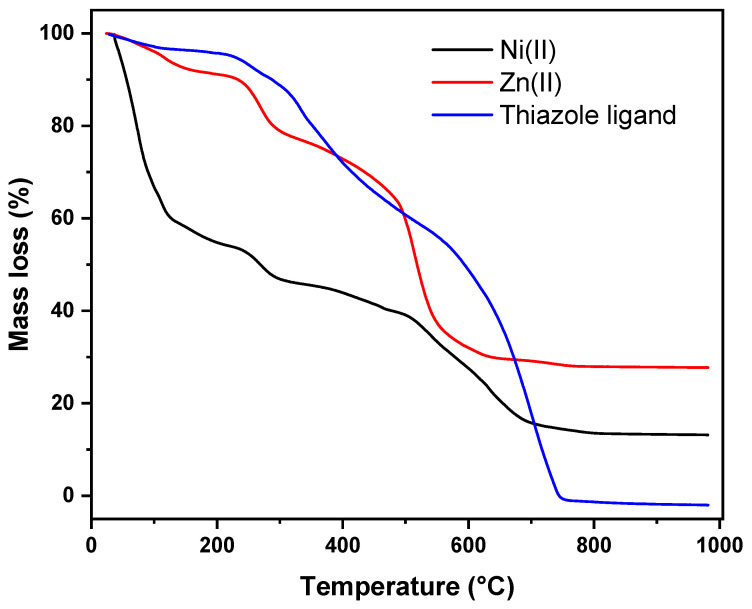
TGA thermograms of thiazole ligand and its complexes.

For the purpose of assessing their stability, the HL Schiff base and its complexes were thermogravimetrically measured in a nitrogen atmosphere. Figure 4 displays the TGA thermogram patterns that were produced. At 46 °C, the Ni(II) complex begins to lose weight from uncoordinated water molecules, while at 54 °C, the water starts to break down from the Zn(II) complex. Within a temperature range of 43–730 °C, the thiazole ligand has three stages. In the first stage, 2.4% (clad. 2.4%) of the mass was lost, which corresponds to the loss of half a mole of water’s moisture, and 16.8% (clad. 16.8%) of the gases 2NH_3_ and CO were decomposed in the second stage. In the last stage, 80.7% (clad. 80.7%) of the weight was eliminated as a fragment [C_15_H_5_BrS] and no mass remnant could be found after the final stage. Three stages were visible on the TGA thermogram for a Zn(II) complex between 54 and 757 °C. A total of 4% (clad. 4%) of the 2H_2_O (lattice water) was lost in the Zn(II) complex’s first stage, 15.7% (clad. 15.8%) of the 4NH_3_ and 2HCl fragments were lost in the second stage and 53% (clad. 54%) of the complex’s mass was lost during the final decomposition because of the loss of [C_20_H_2_Br_2_S_2_O], leaving a residual mass of 27.3%, which was estimated as 26.2%, attributed to Zinc oxide and hydrocarbon(ZnO, 2C_6_H_3_) [39].

The TGA thermogram for a Ni(II) complex shows two stages in the temperature range of 46–767 °C; the first stage shows that the complex is losing 47.6% of its weight (clad. 47.6%), which is represented by gases 2H_2_O, 4NH_3_, 2NO_3_, 2CO, and 2HBr; the second stage occurs with a weight loss of 37.8% (clad. 37.8%), which is associated with the [C_24_H_2_S_2_] fragment. Lastly, metal carbons (Ni, C_6_H_6_) are responsible for the remaining 14.6% of the mass (clad. 14.6%) [39,42].

### 2.9. Antibacterial Activity

**Figure 5:**
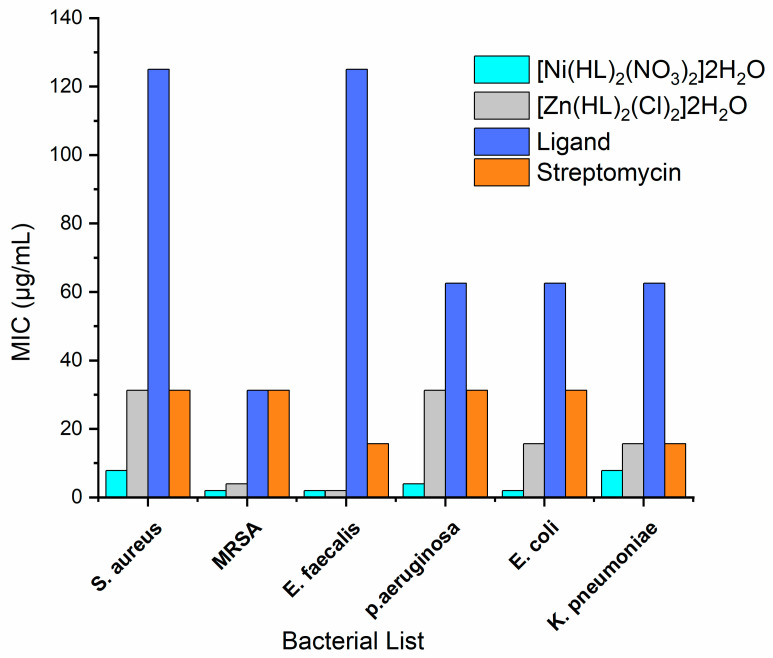
MIC values of thiazole ligand, its complexes and standard streptomycin.

The minimum inhibitory concentration (MIC) values, which ranged from 1.95 to 250 µg/mL are represented in Table 2. This is the first study that evaluates the antibacterial activity of (E)-2-(((5-bromothiazol-2-yl)imino)methyl)phenol and their complexes. All tested compounds showed antibacterial activity with different MIC values, as shown in Table 3 and Figure 5. The Ni(II) complex showed the highest antibacterial activity against selected Gram-positive and Gram-negative bacteria, with an MIC value of 1.95 µg/mL against (*MRSA*, *E. coli*, *E. faecalis*), 3.91 µg/mL against *P. aeruginosa* and 7.81 µg/mL for (*S. aureus*, *K. pneuomoniae*).

In comparison to standard streptomycin, the Ni(II) complex has the greatest activity against all of the bacteria listed in Table 3. In addition, Zn(II) complex exhibited the highest activity compared to standard streptomycin against *E. faecalis*, *MRSA* and *E. coli*, with MIC values of 1.95 µg/mL, 3.91 µg/mL and 15.63 µg/mL, respectively, but MIC values of 15.63 µg/mL, 31.25 µg/mL and 31.25 µg/mL, respectively, were found for streptomycin. On the other hand, the Zn complex shows the same activity of streptomycin against *S. aureus*, *P. aeruginosa* and *K. pneuomoniae*, with MIC values of 31.25 µg/mL, 31.25 µg/mL and 15.63 µg/mL, respectively; also, thiazole ligand shows same activity against *MRSA*, with MIC values of 31.25 µg/mL. The lowest antibacterial activity was shown by the thiazole ligand against *E. faecalis* and *S. aureus*, with an MIC value of 125 µg/mL. In comparison to the ligand N-(4-phenylthiazol-2yl)-2-((2-thiaxo-1,2-dihydroquinolin-3-yl)methylene)hydrazinecarboxamide, the Zn and Ni complexes exhibit excellent results against the stated microorganisms, and this activity is shown to be improved by coordination with the metal ions, such as the Zn complex inhibition of *Pseudomonas aeruginosa* at 0.78 µg/mL and the Ni complex at 1.563 µg/mL, but the ligand at 6.25 µg/mL [43]. At a higher concentration (75 µg/mL), the Schiff base o-vanillidene-2-aminobenzothiazole and its Zn and Ni complexes demonstrated good biological activity against the tested bacteria as compared to the control (ampicillin) [44].

In terms of biological activity, such as antibacterial activity, the ligand and its complexes produce different effects; generally, complexes are found to be more effective at inhibiting bacterial growth than the ligand. The distinction between the ligand and its complexes is the metal ion that was bound to the ligand; chelation theory can be used to explain why complexes are more active. A Schiff base ligand containing an imine group and maybe additional heteroatoms donated groups; these groups coordinate with the metal ion according to the metal ion valent. Due to this coordination, the metal complex’s surrounding electron density and polarity reduce. This enhances the complex’s lipophilicity, allowing for the metal complex to easily permeate through the lipoid layer of bacterial membranes and inhibit bacteria activity by blocking active binding sites in bacterial enzymes. Aside from lipophilicity, there are other factors to consider, such as concentration, solubility, heteroatom type, geometry, coordination sites, dipole moment and stereochemistry [19,27].

### 2.10. Theoretical Study

#### Docking Study

The molecular docking results represent the interaction of the ligand and complexes with the active site of *E. coli* NAD Synthetase (PDB ID: 1WXH), along with streptomycin. The docking score measures the strength of the ligand and protein interaction, where a higher score indicates a stronger interaction [45,46].

The docking scores, type of interaction, RMSD and bond distance are summarized in Table 3. The docked compounds displayed high docking scores, ranging from −5.95 to −7.61 kcal/mol. These high docking score values signify a good interaction between the compounds and the active site of the protein.

In this study, the Ni complex and Zn complex demonstrated better binding affinity (docking score of from −7.61 to −6.98 kcal/mol) against *E. coli* NAD synthetase than the standard drug, streptomycin (−6.29 kcal/mol).

**Figure 6:**
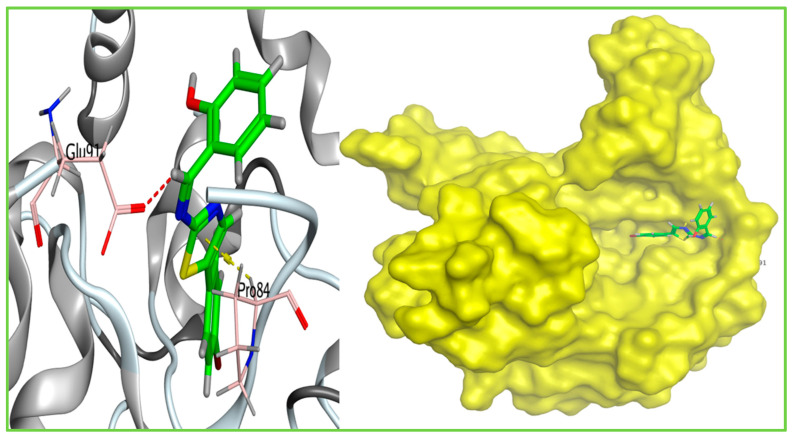
3D image and molecular surface of ligand at the active site of 1WXH.

**Figure 7:**
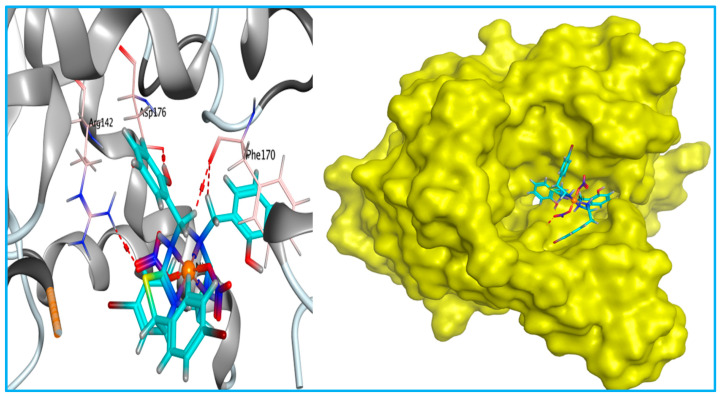
3D image and molecular surface of nickel complex at the active site of 1WXH.

**Figure 8:**
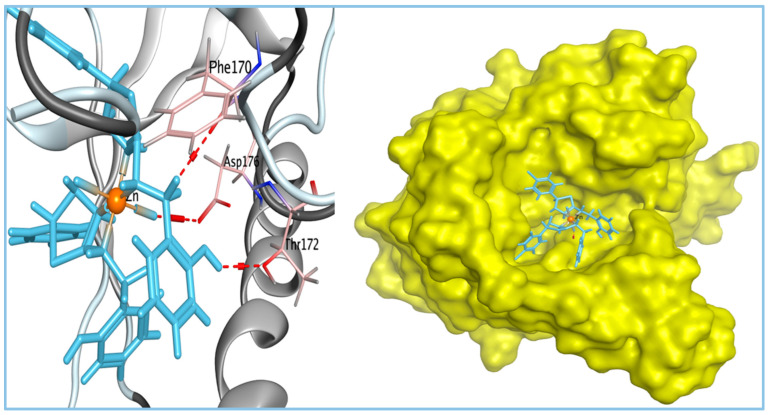
3D image and molecular surface of znic complex at the active site of 1WXH.

The ligand displayed a lower docking score (−5.95 kcal/mol) than streptomycin (−6.29 kcal/mol); it formed only one H-bond with GLU91 and one Pi-H bond with PRO84 (Figure 6). The highest docking score (−7.61 kcal/mol) was recorded by the compound Ni complex. This binding affinity was stabilized by five hydrogen bonds (Figure 7) with amino acid residues PHE170, PHE132, ASP176 and ARG142. The second best docking score (−6.98 kcal/mol) was recorded by the compound Zn complex. This complex formed three hydrogen bonds (Figure 8) with amino acid residues PHE170, THR172, and ASP176.

## 3. Material and Methods

### 3.1. Chemicals

All chemicals and solvents utilized in this study were commercially available and used as-received. 5-(4-bromophenyl)thiazol-2-amine (97%) (C_9_H_7_BrN_2_S) was purchased from Tokyo Chemical Industry Co., Ltd., Tokyo, Japan, Salicylaldehyde (99%) (C_6_H_4_CHO-2-OH) was purchased from LOBA CHEMIE India, N,N-Dimethylformamide with (99.5%) and PEG both were provided by SPECTROCHEM.PVT.LTD.MUMBAI. (INDIA), nickel (II) nitrate hexahydrate (98%) was purchased from Reidel(India)Chemicals Pvt.Ltd and zinc(II) chloride (95%) was purchased from EMPLURA India.

Thin-layer chromatography (TLC) was used to monitor the reaction’s completion, with ethyl acetate and hexane (2:8 volume ratio) as the mobile phase.

### 3.2. Synthesis of Thiazole Schiff Bases

#### 3.2.1. Conventional Method(A)

The thiazole ligand was prepared via the condensation reaction of 3 mmol of 5-(4-bromophenyl)thiazol-2-amine with 3 mmol of salicylaldehyde; these were completely dissolved in 20 mL of ethanol and 3 drops of concentrated sulphuric acid were added. The reaction mixture was refluxed for 6 h and the water bath had a temperature of 80 °C. The greenish-yellow liquid was placed on ice and then filtered, washed with water, dried at room temperature for 24 h, purified by recrystallization from ethanol and placed in a vacuum oven for another 24 h to completely eliminate any remaining solvent traces.

#### 3.2.2. Eco-Friendly Method(B)

In a 100 mL flask, a mixture of (3 mmol) 5-(4-bromophenyl)thiazol-2-amine and (3 mmol) salicylaldehyde were dissolved in a mixture of solvents (water and PEG) with a 1:1 ratio, then refluxed for 3 min in the microwave. Thin-layer chromatography (TLC) with a mobile phase of hexane and ethyl acetate (8:2) was used to monitor the reaction. The same steps mentioned above were applied to the greenish-yellow product to be purified.

HL ligand: yield 89%(A), 90%(B), color: greenish yellow, M.p.196 °C, Anal. Calcd (Found) for C_16_H_11_BrN_2_OS, C:53.50 (53.38), H:3.09 (3.03), N:7.80 (7.74), S:8.92 (8.87), FTIR data (KBr, cm^−1^): *v*(-HC=N- azomethine) = 1604, *v*(>C=N- thiazole) = 1516, *v*(C-S-C) = 753, *v*(C-Br) = 517, *v*(C-O) = 1275, *v*(C=C) = 1496–1455. ^1^H NMR (400 MHz, DMSO-d6) δ/ppm: 10.72 (s, 1H, -OH), 9.70 (s, 1H, azomethine H), 7.22 (s, 1H, thiazole H), 7.14 (d, J = 8 Hz,1H), 7.39 (d, J = 8Hz, 1H), 7.35 (d, J = 8 Hz, 2H), 7.30 (d, J = 8 Hz, 2H), 6.82 (t, J = 8 Hz,1H) and 6.97 (t, J = 8Hz,1H). ^13^C NMR (100 MHz, DMSO-d6) δ/ppm: 166.4(2b), 160.8(1c), 158.8(1), 154.3(4b), 141.9(5b), 132.7(1a), 131.2(5c), 131.0(3c), 129.9(3a,5a), 129.6(2a,6a), 126.7(4a), 123.9(4c), 121.1(2c), 115.7(6c).

#### 3.2.3. Synthesis (E)-2-(((5-bromothiazol-2-yl)imino)methyl)phenol Complexes

In a 50 mL round-bottom flask, (1 mmol) of (E)-2-(((5-bromothiazol-2-yl)imino)methyl)phenol was dissolved in 20 mL of DMF. Next, 0.5 mmol of metal salt ZnCl_2_ or Ni(NO_3_)_2_.6H_2_O was added after being dissolved in 5ml of distilled water, the reaction mixture was basified by adding drops of ammonia solution, the resulting reaction mixture was refluxed for 2 h at (70–80) °C and the product was confirmed by thin-layer chromatography (TLC) (mobile phase hexane: ethyl acetate (8:2)). The coloured product was poured onto ice, filtered and using the same steps as described above for the product to be purified.

Ni(II)complex, yield 55%, color: light brown powder, M.p. >300 °C, Anal. Calcd (Found) for C_32_H_26_Br_2_N_6_NiO_10_S_2_, C: 41.01 (41.03), H: 2.80 (2.78), N: 8.97 (8.53), S:6.84 (6.79). FTIR data (KBr, cm^−1^): *v*(-HC=N- azomethine) = 1658, *v*(>C=N- thiazole) =1521, *v*(C-S-C) = 754, *v*(C-Br) = 516, *v*(C-O) = 1278, *v*(C=C) =1497–1438, magnetic moment = 3.2MB, UV data: (310–364) nm for n–π, 706 nm (*v*_1_) for ^3^A_2g_(F)→^3^T_2g_(F).

Zn(II)complex, yield 70%, color: dark yellow powder, M.p. >300 °C, Anal. Calcd(Found) for C_32_H_26_Br_2_Cl_2_ZnN_4_O_4_S_2_, C: 43.15(43.09), H: 2.94(2.88), N: 6.29(6.22), S: 7.19(7.13), FTIR data (KBr, cm^−1^): *v*(-HC=N- azomethine) = 1658, *v*(>C=N- thiazole) = 1529, *v*(C-S-C) = 754, *v*(C-Br) = 517, *v*(C-O) = 1277, *v*(C=C) =1498–1438. magnetic moment = 0 MB, UV data: (340–387) nm for n–π.

### 3.3. Biological Tests

The thiazole ligand and its complexes were tested against six bacterial strains, with the isolates’ names, kinds, techniques and results illustrated below.

#### 3.3.1. Bacterial Isolates

Six bacterial strains, three gram-positive bacteria (*Staphylococcus aureus* ATCC 25923, *Methicillin-resistant Staphylococcus aureus* ATCC 43,300 and *Enterococcus faecalis* ATCC 29212) and three gram-negative bacteria (*Pseudomonas aeruginosa* ATCC 27853, *Escherichia coli* ATCC 25,922 and *Klebsiella pneumoniae* ATCC 700603) were collected from the Microbiology department, medical college, Aurangabad, India.

#### 3.3.2. Study of Antibacterial Activity

To evaluate the antibacterial activity of thiazole ligands and their complexes, Minimal Inhibition Concentration (MIC) was used [47]. A series of double dilutions ranging from 1.95 to 250 µg/mL were prepared in 1 mL of dimethyl sulfoxide (100% DMSO). Then, 1 mL of nutrient broth was added to each concentration 10 µL of bacterial culture, which was adjusted to the 0.5 McFarland standard, were inoculated into a test tube containing 2 mL of nutrient broth and compounds mixture, respectively. The tubes were incubated at 37 °C for 18–24 h and thereafter observed for turbidity or growth. Streptomycin was used as a standard antibacterial drug (concentration range from 15.63 to 250 µg/mL).

### 3.4. Software Program

The in vitro antibacterial activity of synthetic compounds was compared to that of a reference antibiotic (Streptomycin), and the results are supported by the results of a molecular docking investigation.

#### Molecular Docking Using MOE

The molecular docking was performed using MOE 2022 software. The X-ray crystal structure E. Coli NAD Synthetase (PDB ID: 1WXH) was retrieved from the Protein Data Bank. To begin, the H_2_O molecules were deleted from the sequence using the Sequence Editor window of MOE [48,49]. The second step involved the incorporation of polar hydrogen atoms. Thirdly, Amber14.EHT was used to make the most efficient use of the available energy. In the fourth step, corrections and additions were made to the missing atoms (4103 total). In the fifth step, the site-finder module of MOE was used to assign the active site of the protein (the largest pocket in the protein includes 90 amino acids). The 2D structures of ligand and complexes were made using ChemDraw Professional 16.0. Next, the 3D structures of the ligand and complexes were prepared by MOE software (protonation, partial charges and energy minimization) [50,51].

### 3.5. Instrumentation

Scientific Microwave Synthesis System, 700 W, 2450 MHz (RG 31L, RAGAtech, Pune, India) was utilizesed to synthesize the HL ligand. A CL-726 digital (IndiaMART Member Since, Noida, India) equipment was used to calculate melting points for ligand and their complexes. EuroEA3000 CHNS-O analyzer (EuroVector S.p.A. (EVISA), Milan, Italy) was used to determine the elemental composition. Magnetic susceptibility measurements for all complexes were carried out on solid complex, at room temperature, 24 °C (297 K), using a Sherwood Scientific magnetic susceptibility balance (MSB) Mark I (Cambridge, UK), calibrated with a sealed standard manganese (II) chloride solution. Equation (1) was used to calculate the magnetic moment.

(1) $$\mu \mathrm{eff}=2.84{\surd x}_{m}\times T$$

where μeff is the effective magnetic moment, *x_m_* is the molar susceptibility and T (K) is the absolute temperature.

The coordination bond between metals with ligand and another functional group was recorded by a Nicolet iS10 spectrophotometer (Thermo Scientific Waltham, MA, USA) with an attenuated total reflection (ATR; diamond crystal) accessory used for FTIR analysis. With a total of 32 scans per spectrum and a spectral resolution of 4 cm^−1^, all scans were performed in the 4000–350 cm^−1^ range since the various electronic transitions in the ligand compared with its complexes involving d-d transitions, which were measured by UV/visible spectra acquired using a double-beam UV-Vis spectrophotometer (U-210, Hitachi, Tokyo, Japan) at room temperature, spanning the wavelength range of 800–200 nm in dimethyl sulfoxide/water (2:8 by volume), while all protons and carbon atoms in the thiazole ligand spectra were obtained on a JEOL ECP400 NMR spectroscope (Tokyo, Japan) and were used to record ^1^H and ^13^C NMR spectra at 400 and 100 MHz, respectively.

Thermal analysis for Schiff base and its complexes was performed under dynamic nitrogen gas on a TGA/DSC1 Mettler–Toledo thermogravimeter (Columbus, OH, USA). Samples weighing between 10 and 14 mg were loaded into the TGA alumina crucible and then heated from 25 to 1000 °C at a heating rate of 20 °C min^−1^.

## 4. Conclusions

The synthesis of the thiazole ligand (E)-2-((5-bromothiazol-2-yl) imino) methyl) phenol (HL) was carried out through the use of an eco-friendly strategy and solvent, which resulted in a more efficient and readily available product. This was achieved through the use of microwave-assisted synthesis, which significantly reduced the reaction time from six hours to three minutes. The ligand and its complexes were characterized using various analytical techniques, including magnetic moment, elemental analysis, FTIR, UV-Vis, ^1^H NMR, ^13^C NMR and thermal analysis. The results of the analyses indicated that the ligand interacts as a bidentate ligand, chelating through the two nitrogen atom sites of the thiazole ring and imine group. The magnetic moment value for the nickel complex demonstrated that the Ni complex had an octahedral geometry, which is supported by elemental analysis, FTIR and UV-Vis spectra, as well as thermal studies. In addition, all analyses, with the exception of the magnetic moment, indicated that the Zn complex had an octahedral structure.

Newly synthesized Zn(II) and Ni(II) complexes have potent antibacterial properties, but the Ni(II) complex has shown the strongest antibacterial action against all types of selected bacteria as compared to the Zn(II) complex, ligand and streptomycin, revealing that the Ni(II) complex is a promising antibacterial candidate. This is supported by a docking study showing that the Ni complex demonstrated a better binding affinity and the Nickel complex displayed a higher score than zinc, ligand and standard drugs. The antibacterial activity of the Ni(II) complex could be attributed to several factors, including its structure, and the chelation of the thiazole ligand to the Ni(II) complex likely enhances its antibacterial activity by increasing its stability, facilitating its interaction with bacterial cells and disrupting their normal functioning.

## Figures and Tables

**Table 1:** Analytical data of (E)-2-(((5-bromothiazol-2-yl)imino)methyl)phenol ligand and its complexes.

Compound M. F. [M. wt]	Molecular Formula	Elemental Analyses % Calculated (Found)			
		C	H	N	S
HL,359.24	C_16_H_11_BrN_2_OS	53.50 (53.38)	3.09 (3.03)	7.80 (7.74)	8.92 (8.87)
[Ni(HL)_2_(NO_3_)_2_]2H_2_O, 937.21	C_32_H_26_Br_2_N_6_NiO_10_S_2_	41.01 (41.03)	2.80 (2.78)	8.97 (8.53)	6.84 (6.79)
[Zn(HL)_2_(Cl)_2_]2H_2_O, 890.79	C_32_H_26_Br_2_Cl_2_ZnN_4_O_4_S_2_	43.15 (43.09)	2.94 (2.88)	6.29 (6.22)	7.19 (7.13)

**Table 2:** Minimum inhibitory concentration (MIC in µg/mL) values of thiazole ligand, standard streptomycin and its complexes.

	Gram-Positive Bacteria			Gram-Negative Bacteria		
Compounds	*S. aureus* *ATCC 25923*	*MRSA* *ATCC 43300*	*E. faecalis* *ATCC 29212*	*P. aeruginosa* *ATCC 27853*	*E. coli* *ATCC 25922*	*K. pneumoniae* *ATCC 700603*
[Ni(HL)_2_(NO_3_)_2_]2H_2_O	7.81	1.95	1.95	3.91	1.95	7.81
[Zn(HL)_2_(Cl)_2_]2H_2_O	31.25	3.91	1.95	31.25	15.63	15.63
Ligand(HL)	125	31.25	125	62.5	62.5	62.5
Streptomycin	31.25	31.25	15.63	31.25	31.25	15.63

**Table 3:** Molecular docking results of synthesized compounds along with streptomycin.

	Docking Score (Kcal/mol)	Amino Acid Residue	Type of Bond	RMSD (Kcal/mol)	Bond Length (Å)
Ligand	−5.95	GLU91	H-donor	1.04	3.43
		PRO84	Pi-H		3.76
Ni complex	−7.61	PHE170	H-donor	1.58	2.98
		PHE132	H-donor		4.36
		PHE170	H-donor		3.39
		ASP176	H-donor		3.06
		ARG142	H-acceptor		3.59
Zn complex	−6.98	PHE170	H-donor	1.61	3.08
		THR172	H-donor		2.93
		ASP176	H-donor		3.24
Streptomycin	−6.29	SER48	H-donor	1.86	2.89
		PHE170	H-donor		2.48
		ARG142	H-acceptor		3.02
		ASP52	Ionic		3.14
		ASP52	Ionic		3.51

## Data Availability

No new data were created or analyzed in this study. Data sharing is not applicable to this article.

## Supplementary Materials

The following supporting information can be downloaded at: https://www.mdpi.com/article/10.3390/antibiotics12111634/s1, Figure S1: ^1^H NMR spectrum of (E)-2-(((5-bromothiazol-2-yl)imino)methyl)phenol ligand in DMSO-d6; Figure S2: ^13^C NMR spectrum of (E)-2-(((5-bromothiazol-2-yl)imino)methyl)phenol ligand in DMSO-d6.
