# Supplementary material for: Antimicrobial Activity of Novel Ni(II) and Zn(II) Complexes with (E)-2-((5-Bromothiazol-2-yl)imino)methyl)phenol Ligand: Synthesis, Characterization and Molecular Docking Studies
Source: Antibiotics (Basel). 2023 Nov 17;12(11):1634. doi: 10.3390/antibiotics12111634 (PMC10669075; doi:10.3390/antibiotics12111634)
Supplement: Supplementary file 1 [file antibiotics-12-01634-s001.zip › antibiotics-2674657-supplementary.pdf]

Supplementary Data

Antimicrobial Activity of Novel Ni(II) and Zn(II) Complexes with (E)-2-((5-Bromothiazol-2-yl)imino)methyl)phenol ligand: Synthesis, Characterization and Molecular Docking Studies

Inas Al-Qadsy [1], Waseem Sharaf Saeed,[2] Ahmad Abdulaziz Al-Owais [3], Abdelhabib Semlali,[4] Ali Alrabie [1], Lena Ahmed Saleh Al-Faqeeh,[5] Mohammed ALSaeedy [1], Arwa Al-Adhreai [1] Abdel-Basit Al-Odayni [2] and Mazahar Farooqui [1,*]

[1] Chemistry Department, Maulana Azad College of Arts, Science and Commerce, Aurangabad 431001, India
[2] Department of Restorative Dental Sciences, College of Dentistry, King Saud University, P.O. Box 60169, Riyadh 11545, Saudi Arabia
[3] Chemistry Department, College of Science, King Saud University, P.O. Box 2455, Riyadh 11451, Saudi Arabia
[4] Groupe de Recherche en Écologie Buccale, Faculté de Médecin Dentaire, Université Laval, Quebec, QC G1V 0A6, Canada
[5] Microbiology Department, Dr. Babasaheb Ambedkar Marathwada University, Aurangabad 431004, India

* Correspondence: inas2015228@gmail.com mazaharf@maca.ac.in

NMR spectra:

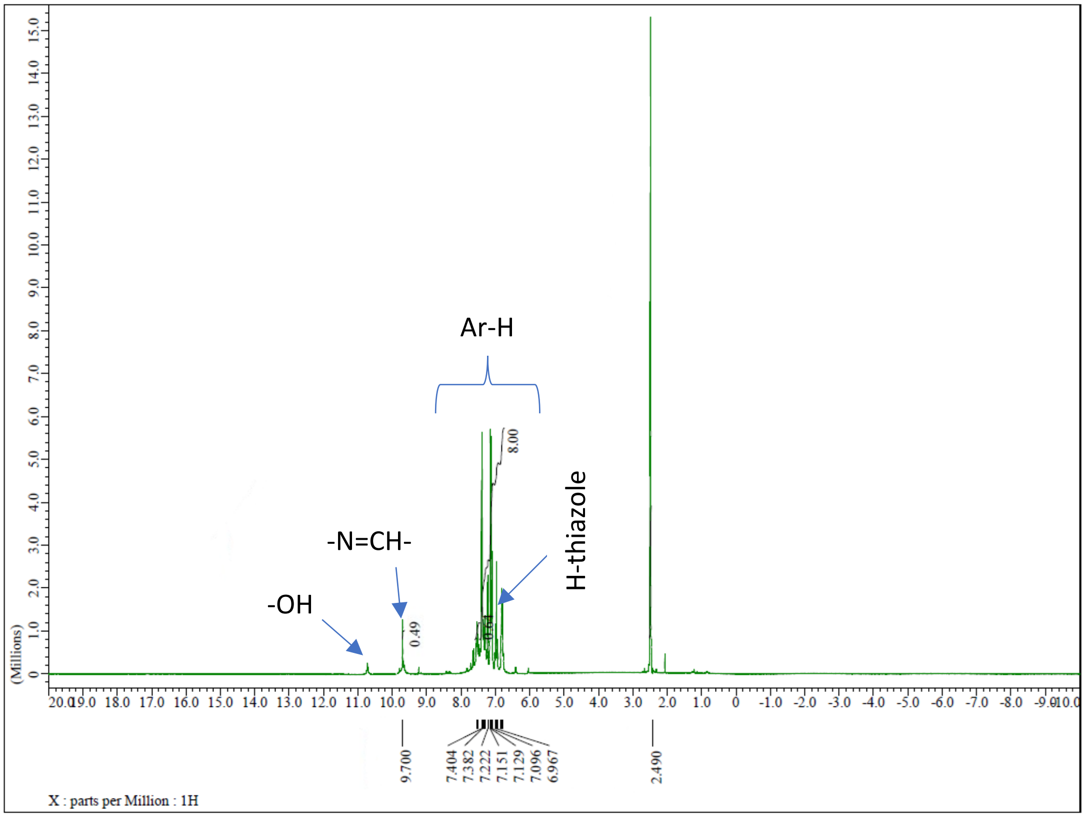

Figure S1. ^{1}H NMR spectrum of (E)-2-(((5-bromothiazol-2-yl)imino)methyl)phenol ligand in DMSO-d_6

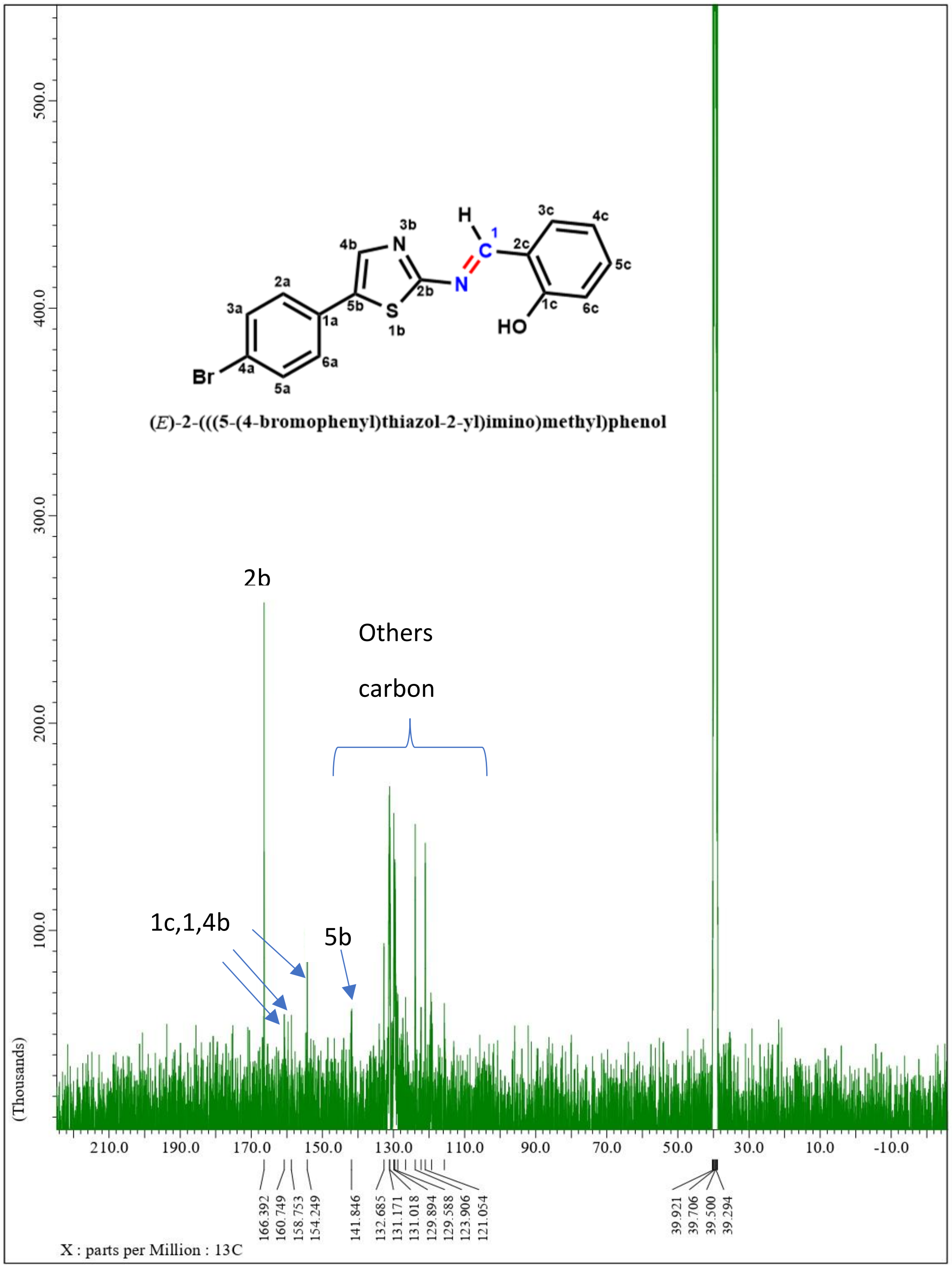

Figure S2. ^{13}C NMR spectrum of (E)-2-(((5-bromothiazol-2-yl)imino)methyl)phenol ligand in DMSO-d6
